# Comparative Analysis of Postictal Delirium Following ECT and Post-Anesthesia Delirium

**DOI:** 10.1192/j.eurpsy.2025.887

**Published:** 2025-08-26

**Authors:** B. Cipion, P. A. White, B. R. Carr

**Affiliations:** 1 College of Medicine; 2 Anesthesiology; 3 Psychiatry, University of Florida, Gainesville, United States

## Abstract

**Introduction:**

Postictal delirium (PD) following Electroconvulsive Therapy (ECT) and Post-Anesthesia Delirium (PAD) are significant postoperative cognitive disturbances often encountered in the post-anesthesia care unit (PACU). While both manifest with cognitive impairments, their etiologies, clinical features, and management strategies differ. Recognizing these distinctions is essential to enhance patient care and outcomes, particularly in critical recovery settings where prompt recognition and intervention are paramount.

**Objectives:**

This study compares delirium’s onset, duration, and course following ECT-induced seizures and general anesthesia. It aims to elucidate the clinical features of PD and PAD and offer evidence-based recommendations for distinguishing and managing these conditions in the perioperative setting.

**Methods:**

A comprehensive literature review was conducted, focusing on studies from 2000 to 2023 sourced from PubMed, MEDLINE, and Cochrane Library. Search terms included “postictal delirium,” “Electroconvulsive Therapy,” “post-anesthesia delirium,” and “perioperative cognitive disorders.” Key variables analyzed included onset, duration, cognitive and behavioral symptoms, associated risk factors, and treatment protocols for both conditions.

**Results:**

The analysis revealed key differences between PD and PAD. PD generally presents immediately after ECT and resolves within minutes to hours, whereas PAD has variable onset, occurring immediately after surgery or several days later, with symptoms lasting hours to days. Cognitive symptoms also differ. PD is characterized by brief confusion and both anterograde and retrograde amnesia, while PAD presents with prolonged confusion, disorientation, and short-term memory impairment. Behaviorally, PD often involves repetitive, patterned, involuntary movements (stereotypies), such as hand flapping and rocking, whereas PAD is characterized by non-patterned agitation, including both voluntary and involuntary movements. PD typically includes fatigue and altered consciousness, while PAD may present with hallucinations, delusions, and significant sleep disturbances. Risk factors for these syndromes also vary. PD is linked to the intensity of the ECT stimulus and pre-existing neurological conditions, while PAD is influenced by factors such as patient age, type of surgery, anesthesia duration, and baseline cognitive status.

**Conclusions:**

PD and PAD share clinical overlap, particularly in cognitive symptoms, but they differ in onset, duration, behavioral patterns, and associated risk factors. PD following ECT is typically brief and marked by stereotyped movements, while PAD presents with prolonged confusion and non-patterned agitation. Accurate differentiation between these conditions is crucial for appropriate diagnosis and management in the PACU setting. Further research is needed to uncover the underlying mechanisms and enhance therapeutic strategies for these syndromes.

**Disclosure of Interest:**

None Declared

